# Evaluation of APTES-Functionalized Zinc Oxide Nanoparticles for Adsorption of CH_4_ and CO_2_

**DOI:** 10.3390/molecules29215219

**Published:** 2024-11-04

**Authors:** Luis A. Montejo-Mesa, Alicia M. Díaz-García, Celio L. Cavalcante, Enrique Vilarrasa-García, Enrique Rodríguez-Castellón, Daniel Ballesteros-Plata, Giselle I. Autié-Castro

**Affiliations:** 1 Laboratory of Bioinorganic, Department of General and Inorganic Chemistry, University of Havana, Havana 10400, Cuba; luismm@unah.edu.cu; 2 GPSA-Group of Research in Separations by Adsorption, Department of Chemical Engineering, Federal University of Ceará, Campus do Pici, Fortaleza 60001, CE, Brazil; celio@gpsa.ufc.br (C.L.C.J.); enrique@gpsa.ufc.br (E.V.-G.); 3 Faculty of Sciences, Department of Inorganic Chemistry, University of Malaga, 29071 Málaga, Spain; daniel.ballesteros@uma.es; 4 Institute of Science and Technology of Materials (IMRE), University of Havana, Havana 10400, Cuba; giselleautie@gmail.com

**Keywords:** ZnONPs@APTES nanoparticles, functionalization, adsorption, separation, CH_4_, CO_2_

## Abstract

ZnO nanoparticles functionalized with APTES were obtained to evaluate their CH_4_ and CO_2_ adsorption at 298 K in a range between 0 and 10 bar. First, ZnO nanoparticles were obtained by a precipitation method and subsequently coated with (3-aminopropyl)triethoxysilane (APTES). As a preliminary study, the results were compared with previously reported naked nanoparticles in order to evaluate the influence of APTES coating on CO_2_ selectivity. UV-Vis, FT-IR spectroscopy, TGA, XRD, TEM/EDX, XPS and N_2_ adsorption at 77 K were used to characterize the evaluated material. It was observed that the amount of gas adsorbed on the surface of the nanostructure was very small in comparison with other materials traditionally used for this purpose but slightly higher than those obtained in naked nanoparticles evaluated in previous studies. The affinity of CO_2_ for the amines groups of the APTES ligand was also discussed.

## 1. Introduction

Zinc oxide (ZnO) is one of the most widely used semiconductors in everyday life; it can be called a multifunctional material due to its unique physical and chemical properties. The hexagonal wurtzite-type crystalline structure takes place in nature, although it may also occur with cubic blende-type structure.

The nanostructured ZnO presents a gap of 3.37 eV at room temperature, with an exciton energy of 60 meV. The strong exciton binding energy (25 meV) and thermal energy at room temperature (26 meV) can ensure efficient exciton emission at the above conditions. As a consequence, this material may be used in photoelectronics [1] and electronic equipment [2], in sensors [3,4,5,6,7], in UV laser [8], as well as in solar cells [9]. Also, it is known that ZnO photoluminescence properties depend on the its crystal size, defects in its crystalline structure and temperature [10].

Other applications in which ZnO has played a fundamental role have been in catalytic processes [11]. On the other hand, the antibacterial, disinfectant and drying properties of zinc oxide allows its use in the production of various types of pharmaceutical products [12,13].

The separation of CH_4_ and CO_2_ from natural gas has generated great interest in recent years. There are different materials capable of achieving this objective with higher or lower effectiveness, such as zeolites, activated carbons and MOFs, among others [14,15,16]. Some types of nanostructures have been evaluated according to their adsorption capacity for CO_2_ and CH_4_, for example: T-type zeolite nanoparticles and amino-functionalized Zr-MOF nanoparticles, which exhibited selective adsorption of CO_2_ over CH_4_ [17,18]. Moreover, metal oxides have been studied for this purpose [19].

In this study, a preliminary evaluation of CO_2_ adsorption by ZnO nanoparticles functionalized with (3-aminopropyl)triethoxysilane (APTES) was carried out. The obtained results were compared with a previous study where the adsorption and separation of CH_4_ and CO_2_ by naked ZnO nanoparticles were discussed [20].

## 2. Results

### 2.1. Synthesis and Characterization of ZnO and ZnO@APTES Nanoparticles

ZnO nanoparticles (ZnONPs) were obtained by a method similar to the one previously reported by Hariharan [21]. The ZnONPs were functionalized with APTES by a post-synthesis procedure (ZnONPs@APTES). The naked and functionalized nanostructures were then characterized using different techniques to provide information on their structural, morphological and surface characteristics.

**Figure 1:**
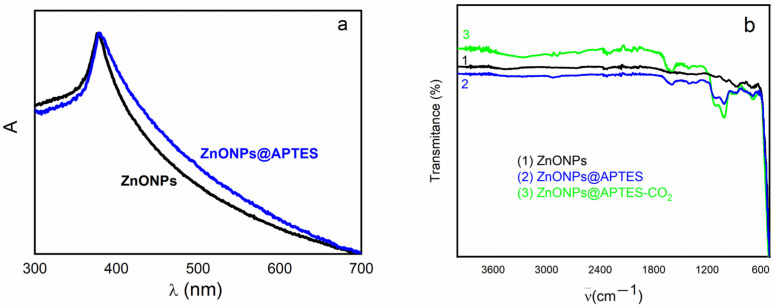
UV-Vis absorption spectra (**a**) and FT-IR (**b**) of ZnO and ZnO@APTES nanostructures. The FT-IR spectrum of ZnO@APTES after CO_2_ adsorption is also shown.

The UV-Vis spectra of both synthesized samples are shown in Figure 1a, suggesting the formation of ZnO. The presence of characteristic bands of APTES on the surface of ZnO may be observed in the FT-IR spectra (Figure 1b).

**Figure 2:**
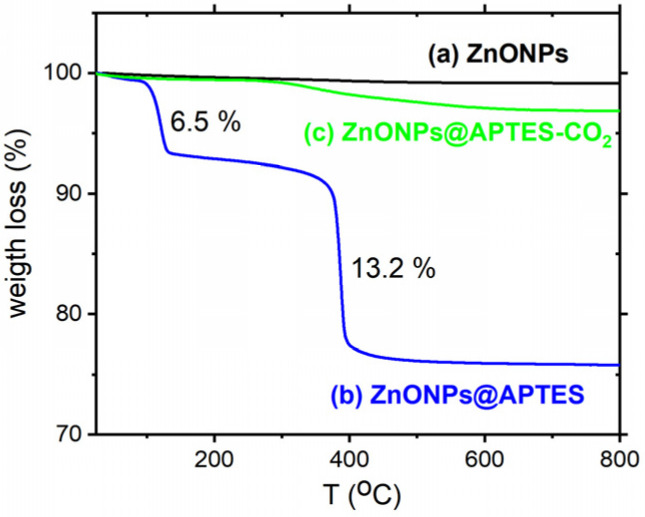
Thermogravimetric curves of (a) ZnONPs, (b) ZnONP@APTES and (c) ZnONP@APTES after CO_2_ adsorption of up to 1 bar.

A thermogravimetric measurement was performed on the naked and functionalized ZnONPs to determine the amount of organic matter in the APTES-coated ZnO (Figure 2). Differences between naked ZnONPs and ZnONPs functionalized with APTES were observed. The ZnONPs@APTES remained stable until 673 K due to the organic coverage. The ZnONPs@APTES curve after CO_2_ adsorption at low pressure exhibits two mass losses, which will be discussed further below.

**Figure 3:**
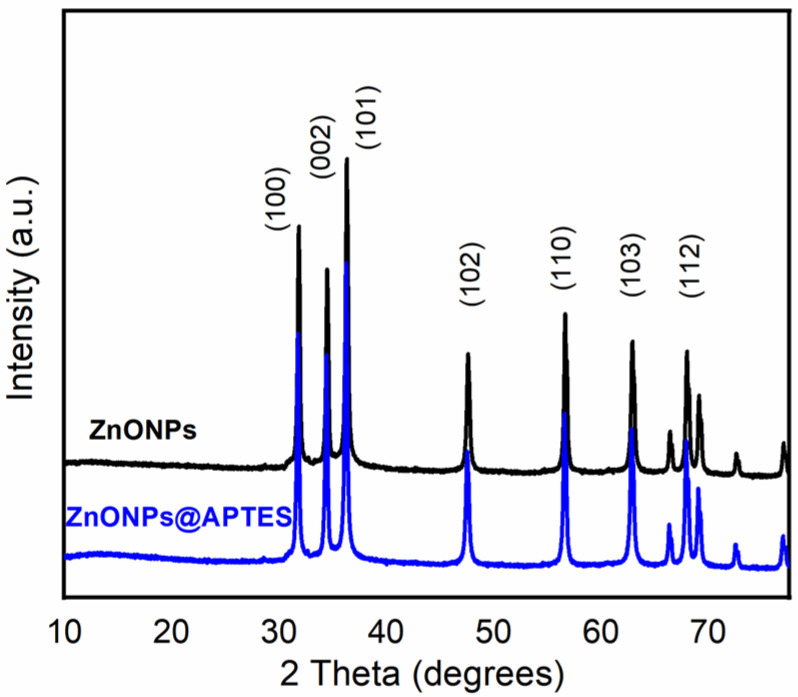
Diffraction patterns of ZnONPs and ZnONPs@APTES nanostructures.

The structural characterization of the synthesized nanostructures was performed by XRD. The obtained XRD patterns of naked and functionalized ZnONPs with APTES are shown in Figure 3.

**Figure 4:**
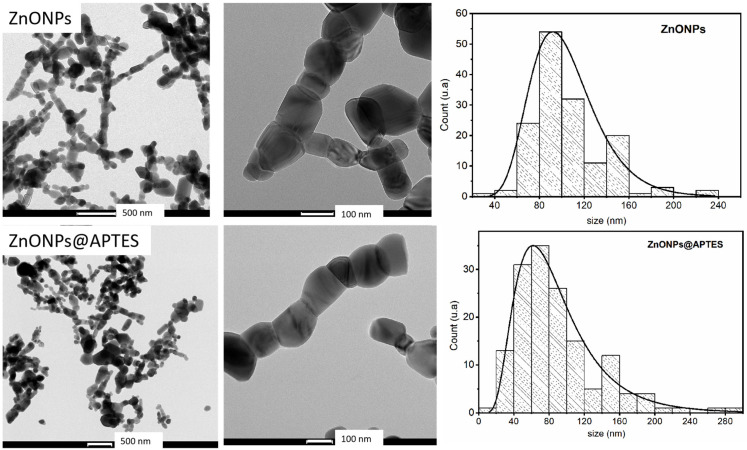
HRTEM images and particle size distributions of ZnONPs [20] and ZnONPs@APTES. Particle distributions of naked ZnONPs and ZnONPs@APTES are also shown.

**Figure 5:**
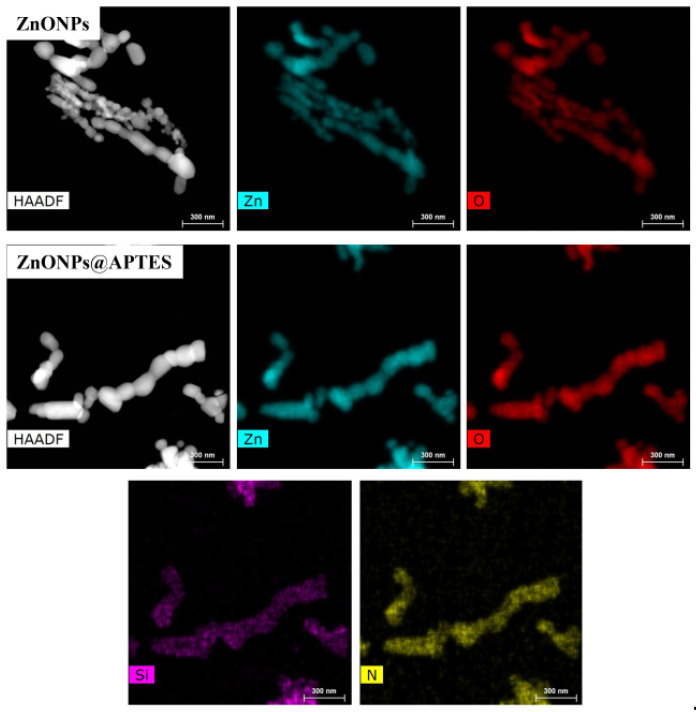
Elemental mappings of ZnONPs and ZnONPs@APTES.

The HRTEM and elemental mapping images for both nanostructures are shown in Figure 4 and Figure 5, respectively.

The weight and atomic percentages of Zn, O, Si and N are summarized in Table 1, with oxygen as the major element, as expected.

**Figure 6:**
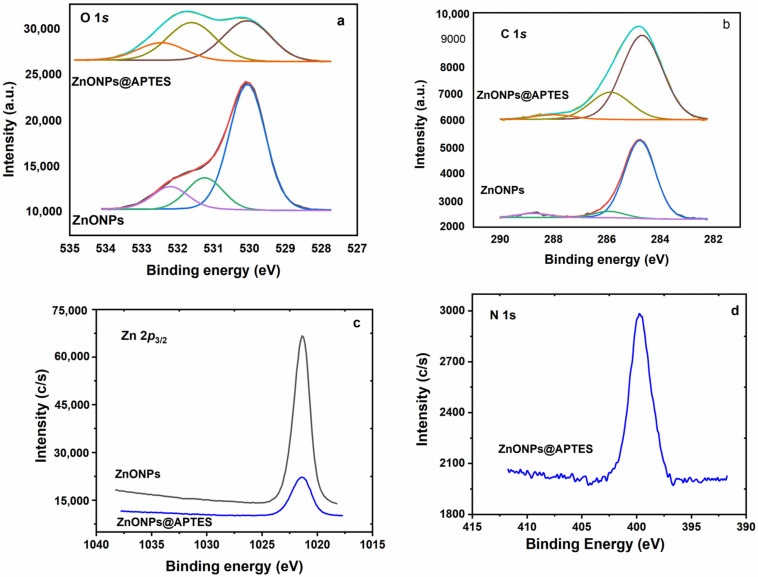
C 1*s* (**a**), O 1*s* (**b**), Zn 2*p*_3/2_ (**c**) and N 1*s* (**d**) core level spectra of the studied samples.

The surface and the chemical state of the constituent element for samples ZnONPs and ZnONPs@APTES were studied by XPS. The high-resolution C 1*s*, O 1*s* and Zn 2*p*_3/2_ core level spectra for both samples, as well as the N 1*s* core level spectrum for the ZnONPs@APTES sample, are displayed in Figure 6. The binding energy values of the constituent elements and the surface chemical composition are shown in Table 2.

### 2.2. Gas Adsorption Experiments for ZnONPs@APTES

**Figure 7:**
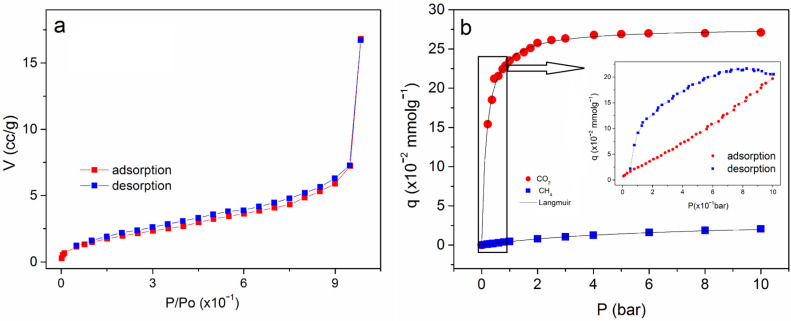
Adsorption isotherms of N_2_ at 77 K (**a**); CO_2_ and CH_4_ at 298 K up to 10 bar (**b**) in the ZnONPs@APTES sample. Inset (**b**): CO_2_ adsorption isotherm up to 1 bar.

The adsorption isotherm of N_2_ at 77 K in the ZnONPs@APTES sample is shown in Figure 7a. From the N_2_ adsorption measurements, the surface area and pore volume were calculated (see Table 3). Also, the CO_2_ experimental adsorption isotherm at 298 K up to 10 bar is shown in Figure 7b. The evaluated material exhibits a low coverage where each active site was able to adsorb only one molecule (monolayer adsorption). Thus, the obtained adsorption isotherms can be interpreted according to the Langmuir model (Figure 7b). From the non-lineal fit, the values of the maximum adsorbed amount (*q_max_*) and the *b_i_* parameter were calculated. The inset of Figure 7b exhibits the CO_2_ experimental adsorption isotherm at 298 K up to 1 bar where a hysteresis loop is observed. Considering the decrease in pore volume after functionalization with subsequent pore clogging, interstices with mesoporous dimensions can be formed where adsorption takes place. The CH_4_ adsorption isotherm at similar conditions was also measured, but no significant adsorption was observed.

Table 4 shows the values of the maximum adsorbed amount for CO_2_ in ZnONPs@APTES (this work) as well as different materials from previous reports at up to 1 bar of relative pressure.

## 3. Discussion

The naked and APTES-coated ZnO nanoparticles have a characteristic band in the UV-Vis spectrum, which allows their identification. An absorption band is observed around 375 nm, which could be assigned to the transition from the valence band to the conduction band (O_2p_→Zn_3d_) (Figure 1a) [22]. This band is exhibited at a shorter wavelength than that of the bulk ZnO, which was reported to arise at 385 nm [23]. It has been previously reported in studies that the absorption maximum can shift to shorter wavelengths by decreasing the particle size [24,25].

The FT-IR spectrum of the ZnONPs@APTES sample is shown in Figure 1b. The presence of APTES was verified by comparing the FT-IR spectra of both samples. The band corresponding to the Zn-O valence vibration (around 450 cm^−1^) can be observed in the spectrum of ZnONPs@APTES, which indicates the presence of ZnO. The broad band towards 3395 cm^−1^ refers to the NH valence vibration (ν_N-H_), while the band located at 1635 cm^−1^ is assigned to the dubbing vibration HNH (δ_H-N-H_) of the free amino groups [24]. The signal that appears at 1110 cm^−1^ corresponds to the Si-O valence vibration (ν_Si-O_) of the silanol groups present as a result of the functionalization. The bands (ν_N-H_, δ_H-N-H_ and ν_Si-O_) confirm the presence of APTES at the surface of ZnONPs.

In addition, CO_2_ adsorption was performed at 298 K up to 1 bar of pressure to evaluate the regenerability and hydrothermal stability. TGA and FT-IR data were collected from the ZnONPs@APTES sample after CO_2_ adsorption. The valence vibration of Si-O (ν_Si-O_) increased in the sample after adsorption, which may be related to the carbamate formation, which is discussed below.

The thermogram obtained for the ZnONPs and ZnONPs@APTES samples is shown in Figure 2. The thermogravimetric curve of ZnONPs@APTES exhibits two stages of weight loss. The first stage occurs at around 115 °C, corresponding to the loss of 6.5% of water adsorbed on the surface of ZnO nanoparticles. The second weight loss (13.2%) takes place at 388 °C, which could be ascribed to the decomposition and degradation of organic matter (APTES) [26]. These results may be related to the analysis of the FT-IR spectrum, which exhibits characteristic bands corresponding to the presence of APTES at the surface of ZnONPs. A thermogravimetric measurement was also carried out after CO_2_ adsorption at low pressures (up to 1 bar) in order to verify the hydrothermal stability. While the material remained stable up to 673 K, the thermal behavior is different from that of the sample before CO_2_ adsorption. The two steps of weight loss may be ascribed to the water molecules as well as gases strongly adsorbed on the surface of ZnONPs@APTES.

The structural characterization of the synthesized nanoparticles was performed by XRD. The obtained XRD patterns of naked and functionalized ZnONPs with APTES are shown in Figure 3. The diffraction peaks are assigned to the (1 0 0), (0 0 2), (1 0 1), (1 0 2), (1 1 0), (1 0 3) and (1 1 2) planes, characteristic of the structure of the hexagonal wurtzite ZnO (P63mc space group) (JCPDS No. 79-2205) [27]. Narrow diffraction peaks are observed in both samples, which suggests good crystallinity [28].

High-resolution TEM images of both samples are shown in Figure 4, where particles with a quasi-spherical or hexagonal shape may be observed. The average particle sizes of the uncovered ZnONPs and ZnONPs@APTES are 91 and 62 nm, respectively. After grafting silanoid compounds onto the surface, the morphology of nanostructures was preserved. The decrease in particle size of ZnO@APTES may be ascribed to the synthesis method used. These particles agglomerated to form rod-like structures. The elemental mapping of the ZnONPs@APTES sample (Figure 5) shows the distribution of Zn, O, Si and N, which are well dispersed in all areas of the particles, indicating that APTES is incorporated uniformly on the entire surface of the ZnO particles. The weight and atomic percentage of Zn, O, Si and N are summarized in Table 1, with oxygen as the major element, as expected.

The presence and surface concentrations of N, O, Si and Zn were studied by XPS analysis. The C 1*s* spectrum of the ZnONP samples can be decomposed in three contributions at 284.8, 285.9 and 288.8 eV. The more intense contribution at 284.8 eV is assigned to adventitious carbon, and the other weak contributions at 285.9 and 288.8 eV are assigned to C-OH and carbonate groups, respectively. The C 1*s* spectrum of the sample functionalized with APTES also shows three contributions. However, the contribution at 285.9 eV exhibits a relative intensity much more intense than the naked ZnONPs, which could be ascribed to the presence of C-N bonds of the amino group [29]. The high-resolution O 1*s* core level spectrum of ZnONP_S_ can be decomposed in three contributions at 530.1, 531.2 and 532.2 eV, assigned to the lattice oxygen of ZnO, the Zn-OH group and carbonate, respectively [30]. Upon functionalization with APTES, a contribution at 531.6 eV with a high relative intensity is observed due to the presence Zn-O-Si bonds (Figure 6b), confirming the grafting of APTES on the ZnO nanoparticles. Figure 6c also includes the Zn 2*p_3/2_* spectra for both solids. The observed binding energy values are similar and typical of ZnO [30], but the intensity of this signal in the case of ZnONPs@APTES is much less intense due to APTES covering the ZnO nanoparticles. In fact, the atomic concentration of Zn decreases from 37.25% for the naked ZnONPs to 11.38% for the functionalized ZnONPs@APTES. Finally, the high-resolution N 1*s* spectrum for ZnONPs@APTES shows a single peak at 399.7 eV. This value is similar to those found in the case of materials coated with APTES [29,30].

The chemical surface composition (Table 2) shows a clear increase in the carbon content upon functionalization from 20.02 to 39.31%. The presence of N and Si was detected and the N/Si atomic ratio of 7.16/8.84 = 0.81 is relatively close to the theoretical values of 1.00. In summary, the formation of a covalent bonding between ZnO nanoparticles and APTES was verified by XPS.

The adsorption isotherm of N_2_ at 77 K in the ZnONPs@APTES sample is shown in Figure 7a. The isotherms were of type II with a hysteresis loop H3 according to the IUPAC classification [31]. This type of isotherm is characteristic of nonporous or macroporous adsorbents and represents adsorption in monolayer or multilayer forms without restrictions. The specific surface area value obtained using the BET method [32] was similar to the naked NPs (Table 3). The pore volume value decreased slightly after functionalization, which could be ascribed to the partial block of the porosity by APTES molecules (Table 3). The *C* constant was higher in the functionalized material, which indicates a stronger interaction with the adsorbate due to the silanol groups of the APTES contribution.

The CO_2_ experimental adsorption isotherm at 298 K up to 10 bar in the ZnONPs@APTES sample is revealed in Figure 7b, showing a Langmuir type I behavior. The maximum adsorbed amount was 0.277 mmol g^−1^, slightly higher than the previously reported value for naked ZnONPs (0.240 mmol g^−1^) [20]. A Langmuir fit is also shown in Figure 7 with maximum adsorbed quantity (*q_max_*) values and the *b_i_* parameter, related to the adsorbent–adsorbate interaction, estimated at 0.277 ± 0.001 mmol g^−1^ and 5.847 ± 0.218 bar^−1^, respectively. In both cases, regression coefficients (*r*^2^) were higher than 0.978, indicating that the Langmuir equation represents the experimental values with good precision. Therefore, only one molecule was adsorbed at each site; the sites were energetically homogeneous and there was no interaction between the adsorbate molecules (lateral interactions) [31]. CO_2_ adsorption took place especially on APTES molecules dispersed on the surface, which also explains why CH_4_ was not adsorbed. The amines groups located on the external surface of the nanoparticles were the energetic active sites where CO_2_ was adsorbed due to the high affinity of CO_2_ molecules by these groups. On the other hand, the studied material did not adsorb any significant quantity of CH_4_ (it falls within the error of the balance), which could probably be ascribed to the weak interaction of the CH_4_ molecule with amines groups of APTES present on the ZnO nanoparticles after functionalization.

As can be seen, the amount of gas adsorbed on the surface of the nanoparticles was very small compared to the materials traditionally used for this purpose (zeolites, clays and active charcoals) [33,34,35]. This behavior may be due to the low values of surface area and pore volume obtained by the adsorption of N_2_ at 77 K. To improve these results, the functionalization with ligands capable of increasing the surface areas of the nanostructures evaluated would be beneficial. In that sense, it would be possible to improve the adsorbate–adsorbent affinity with the incorporation of higher-energy sites and consequently upgrade the amount adsorbed.

Furthermore, the Henry constant, K, for CO_2_ on ZnONPs@APTES was calculated at very low relative pressures. The K value of 0.277 mmol g^−1^ bar^−1^, obtained between 0 and 0.06 bar, assesses the adsorption affinity at low surface coverages. Since CO_2_ molecules interact with the amine groups of APTES, as will be discussed below, the relatively higher K value reflects this specific interaction. For comparison, the Henry constant for CH_4_ was calculated as 0.0051 mol g^−1^ bar^−1^, indicating a lower adsorption affinity due to the non-polar nature of CH_4_ molecules and the limited interaction with ZnONPs@APTES.

The adsorption of CO_2_ and the null adsorption of CH_4_ by the nanoparticles functionalized with APTES could be due to the fact that the APTES amino groups (which are responsible for the capture) are selective to CO_2_. Since the acidic CO_2_ molecules interact with the basic surface groups, the formation of ammonium carbamate species occurs under anhydrous conditions and ammonium bicarbonate species in the presence of water [36,37]. Therefore, given the activation conditions previously explained, with the subsequent absence of water, the formation of ammonium carbamate would be favored. The advantage of using coatings that have amino groups in their structure is that they could provide selectivity in process of CO_2_/CH_4_ separation.

**Figure 8:**
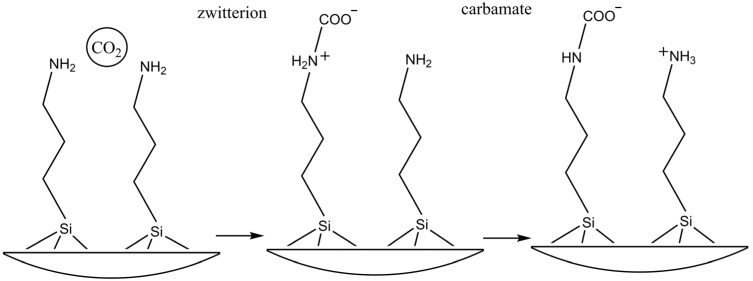
Representation of the interaction mechanism between CO_2_ molecules and amino groups present at the ZnONPs@APTES surface.

The adsorption of CO_2_, as well as the null CH_4_ adsorption by the nanostructures functionalized with APTES, is related to the fact that the amino groups present in APTES (which are responsible for the capture) are selective for CO_2_, since the acidic CO_2_ molecules interact with the basic surface groups. Thus, the formation of ammonium carbamate species in anhydrous conditions and ammonium bicarbonate species takes place (Figure 8) [38,39,40]. The advantage of using coatings with amino groups in their structure is providing selectivity to the separation process of these types of gases.

Amino groups have been of great interest in organic functionalization due to their high reactivity and selectivity towards CO_2_ and other acid gases. The mechanism of this reaction was proposed by M. Caplow 1968 [41] and reintroduced by Danckwertz in 1979 [42], who described the reaction between CO_2_ and amino groups (primary and secondary) through the formation of an unstable amphoteric salt (zwitterion) followed by deprotonation of the base. The reaction mechanism is defined in two stages:

1. Nucleophilic attack of the amino group on the carbon of CO_2_ and formation of the intermediate zwitterion (R_1_R_2_NH^+^COO^−^).

R_1_R_2_NH + CO_2_^®^ R_1_R_2_NH + COO^−^ (1)

2. Acceptance of the proton by a base. Under anhydrous conditions, this function is performed by an adjacent amino group (primary or secondary) leading to the formation of the carbamate (R_1_R_2_NCOO^−^).

R_1_R_2_NH + COO^−^ + Base (B) ^®^ BH^+^ +R_1_R_2_NCOO^−^ (2)

The deprotonation of the zwitterion can be favored by the presence of any molecule of a basic nature. Therefore, the maximum CO_2_/N ratio theoretically achievable is 0.5 in anhydrous conditions (one molecule of CO_2_ for every two amino groups, or active sites).

## 4. Materials and Methods

### 4.1. Materials

Zinc acetate dihydrate (Zn(CH_3_COO)_2_·2H_2_O, 98%) and oxalic acid dihydrate (H_2_C_2_O_4_·2H_2_O, 99%) were supplied by Merck (Darmstadt, Germany). Ethanol, acetone and nitric acid (HNO_3_, 69%) were purchased from Panreac (Castellar del Vallès, Spain). (3-Aminopropyl)triethoxysilane 99% (APTES) was supplied by Fluka AG, Chem. (Buchs, Switzerland). All solvents were used without pretreatment or further purification and were purchased under the categories “pure for synthesis” or “pure for analysis”.

### 4.2. Synthesis of ZnO Nanoparticles (ZnONPs)

ZnO nanoparticles were prepared by slightly modifying the method used by Hariharan [21]. A total of 1.09 g of Zn(CH_3_COO)_2_·2H_2_O was dissolved in 30 mL of ethanol at 60 °C. The salt was dissolved completely in 10 min. Simultaneously, 1.26 g of oxalic acid was dissolved in 20 mL of ethanol at 50 °C. The oxalic acid solution was slowly added to the hot ethanolic solution of zinc acetate, maintaining the stirring of the reaction mixture. A white gel was formed, which was dried at 80 °C for 20 h. The xerogel was calcined at 800 °C for 2 h to obtain the ZnO nanostructures [20].

### 4.3. Synthesis of ZnO Nanoparticles Functionalized with APTES (ZnONPs@APTES)

A total of 1.5 g of the previously synthesized ZnONPs were dispersed in 50 mL of distilled water. HNO_3_ (2 mol L^−1^) was added dropwise under magnetic stirring, and subsequently, NaOH (1 mol L^−1^) was added until a pH = 6.5 was reached. The dispersion was kept under constant stirring for one hour. Subsequently, 1 mL of APTES was added. The pH after the addition was maintained in the range between 8.9 and 9.2. The reaction mixture was left under stirring for 24 h. The particles were filtered and washed with ethanol and acetone. The powder was dried at 60 °C under vacuum.

### 4.4. Spectroscopic, Morphological and Thermogravimetric Characterization of ZnONPs and ZnONPs@APTES

UV-Vis measurements were performed using an Amershan-Biosciences Ultrospec 2100 pro Spectrophotometer (Chicago, IL, USA) with the Wavescan auxiliary software version 2.4.R33783 (Eddyfi Technologies, Québec, QC, Canada). For the recording of the spectra, quartz cuvettes with an optical step of 1 cm were used. The sample was dispersed in dimethylsulfoxide (DMSO) with ultrasound for 10 min, prior to the registration of the spectrum.

FT-IR spectra were recorded in an FT-IR WQF-510 Spectrophotometer (Beijing Rayleigh Analytical Instrument Corporation, Beijing, China), using pellets in KBr. The FT-IR spectra were registered between 400 and 4000 cm^−1^.

Thermogravimetric analysis (TGA): The rate of change in the material weight as a function of temperature was continuously recorded while heating the sample in the range between 30 and 800 °C at a constant sweep speed (5.0 °C/min). The thermogram was obtained on a NETZSCH STA 409 PC/PG (Selb, Germany). Aluminum oxide was used as a reference.

X-ray powder diffraction pattern of the sample was taken on a Rigaku diffractometer (Akishima, Japan), Miniflex model, with Bragg–Brentano geometry using a monochromatic CuKα radiation (λ = 1.5408 Å). The monochromator was operated at 35 Kv and 25 mA. The samples were scanned in the range 2θ = 2–80°. ZnONPs@APTES XRD data were refined by the Rietveld method [43].

An equipment TALOS F200x (Thermo Fisher Scientific, Waltham, MA, USA) was employed to analyze the morphology by High-Resolution Transmission Electron Microscopy (HRTEM), also operating in STEM mode (Scanning Transmission Electron Microscopy), and the microanalysis was carried out with an EDX Super-X system provided with 4 X-ray detectors and an X-FEG beam. The determination of the particle size for the ZnO nanoparticles was carried out using the ImageJ version 1.38x software. The particle size data were fitted assuming a logarithmic probability density function. The adjustment was based on a count of 150 individual particles.

XPS studies were carried out on a Physical Electronics Spectrometer (PHI Versa Probe II Scanning XPS Microprobe, Physical Electronics, Chanhassen, MN, USA) with monochromatic X-ray Al Kα radiation (200 μm, 100 W, 20 kV, 1486.6 eV) and a dual beam charge neutralizer. XPS spectra were analyzed using the PHI SmartSoft-VP 2.10.4.1 software and processed using the MultiPak 9.3 package. The binding energy values were referenced to the adventitious carbon C 1*s* signal (284.8 eV).

### 4.5. N_2_, CO_2_ and CH_4_ Adsorption Experiments

N_2_ adsorption–desorption isotherms were collected at 77 K and *P/P_0_* = 0.01–0.99 using Accelerated Surface Area and the Porosimetry system (ASAP 2050 model from Micromeritics, Norcross, GA, USA). Sample activation was carried out at 250 °C under vacuum for 12 h. The specific surface area of all materials was evaluated using the Brunauer-Emmett-Teller (BET) model [32]. In addition, Accelerated Surface Area and the Porosimetry system ASAP 2020 model from Micromeritics were used to obtain the CO_2_ adsorption-desorption isotherms at 298 K and *P/P_0_* = 0.01–0.99.

The CH_4_ and CO_2_ adsorption measurements were carried out using a Rubotherm magnetic suspension balance (Bochum, Germany). The monocomponent isotherms of CH_4_ and CO_2_ were carried out at 298 K in the pressure range of 0–10 bar. Samples were degassed prior to obtaining the adsorption isotherms at 300 °C for 12 h using a heating ramp of 2 °C min^−1^ until the final temperature.

The equilibrium data determination consisted of exposing the sample to successive increases in pressure after the degassing stage, in which the mass variations were quantified in relation to the pressure, until the equilibrium was reached. The equilibrium condition established was a mass variation of less than 0.1 mg for 30 min. From the recorded mass variation, the amount of gas adsorbed can be calculated using Equation (3):

(3) $$m_{ex}\left(P,T\right)=\Delta_{m}\left(P,T\right)+\left(V_{B}+V_{s}\right)*\rho \left(P,T\right)$$

where ∆*m* is the mass variation, *m_ex_* is the amount of gas adsorbed in excess and [(*V_B_* + *V_S_*)·*ρ_g_*] is the buoyancy effect, and *V_B_* is the volume of balance components, *V_S_* is the volume of the sample solid and *ρ_g_* is the adsorbate density [44].

The adjustment of the CO_2_ isotherms was made using the non-linear Langmuir model according to Equation (4):

(4) $$q=\frac{q_{\max}b_{i}\;P}{1+b_{i}P}$$

where q_max_ is the amount adsorbed at infinite pressure or the amount adsorbed at saturation (mmol g^−1^), and bi is related to the adsorbent–adsorbate affinity (bar^−1^).

## 5. Conclusions

The ZnONPs@APTES material was obtained in two steps: ZnONPs were synthesized from precipitation by hydrolysis of zinc acetate, and functionalized with APTES in the second step. The obtained ZnONPs@APTES nanostructures were characterized by UV-Vis, FT-IR spectroscopy, TGA, XRD, TEM/EDX, XPS and N_2_ adsorption at 77 K. The grafting of APTES onto ZnO nanoparticles was clearly evidenced by XPS. The adsorption isotherm of CO_2_ at 298 K was obtained up to 10 bar, showing a maximum adsorbed amount of 0.28 mmol g^−1^. No significant quantity of methane adsorption was obtained, probably due to the fact that the APTES amino groups are more selective to CO_2_ molecules. The results herein exposed show that functionalization in nanoparticles is a useful alternative to improve the affinity of CO_2_ with functional groups of organic nature.

## Figures and Tables

**Table 1:** Total weight and atomic percentage of each element present in the samples, obtained from EDX analysis.

Elements/Series	Weight (%)		Atomic (%)	
	ZnO	ZnONPs@APTES	ZnO	ZnONPs@APTES
Zn/K-series	70.9	59.1	37.3	23.5
O/K-series	29.1	20.5	62.7	33.3
N/K-series	-	0.3	-	0.6
Si/K-series	-	0.7	-	0.6
C/K-series	-	19.4	-	41.9

**Table 2:** Binding energy values in eV of the constituent elements of the studied samples, and surface chemical composition (in atomic concentration %).

	Sample	C 1*s*	N 1*s*	O 1*s*	Si 2*s*	Zn 2*p*_3/2_
**BE (eV)**	ZnONPs	284.8 (88)		530.1 (69)	-	
		285.9 (7)	-	531.2 (17)		1021.3
		288.8 (5)		532.2 (14)		
	ZnONPs@APTES	284.8 (73) 285.9 (23) 288.0 (4)	399.7	530.1 (42) 531.6 (40) 532.4 (18)	153.1	1021.4
**At. conc. (%)**	ZnONPs	20.0	-	42.7	-	37.2
	ZnONPs@APTES	39.3	7.2	32.3	8.8	11.4

**Table 3:** Specific surface area and pore volume in samples of ZnONPs and ZnONPs@APTES obtained from BET equation.

Sample	Surface Area (m^2^g^−1^)	Pore Volume (cm^3^g^−1^)	C
ZnONPs [19]	7	0.018	2.11
ZnONPs@APTES	8	0.011	27.4

**Table 4:** Maximum adsorbed amount in mmol g^−1^ at 1 bar of ZnONPs@APTES, ZnONPs, amino-Zr-MOF and T-type nanoparticles zeolite as previously reported.

Sample	$q_{maxCO_{2}}$ (mmol g^−1^)	$q_{maxCH_{4}}$ (mmol g^−1^)	Reference
amino-Zr-MOF	2.860	-	[17]
T-type nanoparticles zeolite	3.940	0.720	[18]
ZnONPs	0.240	0.034	[20]
ZnONPs@APTES	0.277	0.019	This work

## Data Availability

Data are contained within the article.
